# Editorial: Spotlight on the traditional medicine in prevention and treatment of diabetes in the aging population

**DOI:** 10.3389/fmed.2024.1478185

**Published:** 2024-09-13

**Authors:** Lai Jiang, Hao Chi, Xuancheng Zhou, Jingyi Tang, Jieying Zhang, Qinhong Zhang, Shenbin Liu, Jiangang Shen, Guanhu Yang

**Affiliations:** ^1^Clinical Medical College, Southwest Medical University, Luzhou, China; ^2^First Teaching Hospital of Tianjin University of Traditional Chinese Medicine, Tianjin, China; ^3^National Clinical Research Center for Chinese Medicine Acupuncture and Moxibustion, Tianjin, China; ^4^Heilongjiang University of Chinese Medicine, Harbin, China; ^5^Institute of Brain Science, Fudan University, Shanghai, China; ^6^School of Chinese Medicine, Li Ka Shing Faculty of Medicine, The University of Hong Kong, Hong Kong, Hong Kong SAR, China; ^7^Department of Specialty Medicine, Ohio University, Athens, OH, United States

**Keywords:** diabetes, Chinese medicine, acupuncture, treatment, biomarkers

## 1 Introduction

As the global population ages, diabetes has become a growing public health problem. According to the World Health Organization, diabetes affects approximately 425 million people globally, with a particularly high prevalence among the elderly population (1). Diabetes is not only a chronic disease, but its complications pose a serious threat to patients' quality of life (2, 3). In this context, it is particularly important to explore and evaluate the application of traditional medicine in the prevention and treatment of diabetes. This Research Topic aims to provide insights into the potential and challenges of traditional medicine in the management of diabetes in the elderly. We have brought together a series of high-quality research findings covering a wide range of aspects such as diabetic foot problems, neurological complications, and the roles and mechanisms of traditional Chinese medicine in the management of diabetes in the elderly. These studies not only provide empirical support for traditional medicine in diabetes management, but also offer new perspectives on our understanding of its deeper mechanisms of action.

The mission of this editorial is to distill and reflect the best of the scholarship presented in this Research Topic and to present readers with a comprehensive picture of the application of traditional medicine in the treatment of geriatric diabetes. We are confident that these selected studies will shed new light on the field of diabetes care and promote in-depth thinking and innovative practice of comprehensive treatment programs for geriatric diabetes.

## 2 Diabetic foot problems

Diabetic foot problems, especially diabetic foot ulcers (DFUs) and infections, are common complications in elderly diabetic patients, seriously affecting their quality of life, and prognosis (4). The included articles focus on the use and effectiveness of herbal medicine in the treatment of diabetic foot problems. A prospective study by Zhao et al. revealed the potential of Chinese medicine Shengji ointment combined with the pineapple protease (bromelain) in promoting healing of diabetic foot ulcers, and identified key risk factors for healing, including plantar ulcer location, low hemoglobin levels, high body mass index (BMI) and high creatinine levels. The significance of this study is that it provides a valuable predictive model to assist healthcare professionals in assessing treatment efficacy and personalizing treatment strategies for each patient and may help to improve patient prognosis by integrating herbal treatments into routine clinical practice. The study by Yang G. et al. compared the efficacy and cost-effectiveness of Fufang Huangbai Fluid hydropathic compress (FFHB) with conventional antimicrobial calcium alginate wound dressing (ACAWD) in the treatment of diabetic foot infections. It was found that FFHB had significant advantages in promoting wound healing, inhibiting bacterial proliferation, and reducing local inflammation and edema, and showed greater cost-effectiveness compared with ACAWD, providing a safe and cost-effective pharmacological treatment option for mild diabetic foot infections. These studies not only demonstrate the potential of TCM in the treatment of diabetic foot complications, but also provide a scientific basis for future clinical practice.

## 3 Diabetic neurological complications

Diabetic neurological complications, including diabetes-associated cognitive decline (DACD) and diabetic peripheral neuropathy (DPN), are common problems among older diabetic patients, severely affecting their cognitive function and ability to perform daily activities (5). A systematic review and meta-analysis by Su et al. assessed the efficacy and safety of combined Chinese and Western medicine in the treatment of DACD, showing significant advantages of combined Chinese and Western medicine in improving glycemic control and cognitive function. The study by Yu et al. explored the mitigating effects of bergenin on high glucose-induced neuroinflammatory injury at the molecular level, using zebrafish, mouse microglial cell line (BV2 cells) and rat models for evaluation. The results showed that bergenin redirects glucose metabolism, attenuates inflammatory responses, and prevents high-glucose-induced neuronal damage through PPAR-γ/NF-κB pathway intervention, providing new perspectives and potential therapeutic strategies for the treatment of diabetes associated cognitive impairment (DACI). The studies by Zhang X. et al. and Fu et al. assessed the efficacy and application prospects of acupuncture and TCM in the treatment of DPN through systematic evaluation and evidence mapping methods, respectively. These studies provide new perspectives for understanding the mechanisms of diabetic neurologic complications and offer diverse therapeutic strategies for clinical management.

## 4 Role and mechanism of traditional Chinese medicine in the treatment of geriatric diabetes mellitus

Traditional Chinese medicine (TCM) plays an important role in the treatment of geriatric diabetes mellitus, and its multi-targeted and individualized therapeutic features provide new therapeutic options for geriatric diabetes mellitus patients. The study by Lei et al. delved into the pharmacodynamic components and molecular mechanisms of Yuquan pill in the treatment of type 2 diabetes mellitus (T2DM) through a multi-omics analysis, providing a scientific basis for understanding the role of TCM. It was found that Yuquan pill was able to reduce the levels of triglycerides, cholesterol, nitric oxide and malondialdehyde in T2DM rats, as well as increase the levels of high-density lipoprotein cholesterol, and had a protective effect on the liver and kidney. Zhang Q. et al. systematically reviewed the clinical application of TCM in the treatment of geriatric diabetes mellitus, pointed out the shortcomings of the current study, and provided guidance for future study design. Yang X. et al.'s study, on the other hand, explored the role of TCM in the improvement of clinical symptoms and glycemic control from the perspective of the clinical characteristics and pathogenesis of geriatric diabetes mellitus, and emphasized the important value of TCM in the management of geriatric diabetes mellitus. value of TCM in the management of geriatric diabetes mellitus. Together, these studies advance the understanding of the roles and mechanisms of TCM in the management of geriatric diabetes and provide a wealth of information and guidance for clinical practice.

## 5 Conclusion and outlook

Overall, the studies included in this Research Topic profoundly reveal the multifaceted role of traditional medicine in the management of geriatric diabetes, particularly in the management of diabetic foot problems and neurologic complications. Through in-depth analyses of the pharmacodynamic components of herbal compounds, the clinical effects of traditional treatments, and the potential mechanisms of TCM for glucose regulation and complication prevention, these studies provide valuable insights into personalized treatment strategies for geriatric diabetes. By mapping out a comprehensive picture of traditional medicine in the treatment of diabetes and understanding how these treatments interact with a patient's specific condition, we are able to design more effective and personalized treatment plans for older patients with diabetes. It is important to note that clinical translation of these findings and a deeper understanding of the biological mechanisms are critical to their successful application to practical treatment strategies. Therefore, future work should include further clinical trials and mechanistic studies to confirm the actual effectiveness and application potential of these traditional treatments.

In conclusion, by deeply exploring the therapeutic potential of traditional medicine and combining it with modern medicine, we are expected to significantly improve the effectiveness of geriatric diabetes treatment and open up new therapeutic avenues. This approach not only demonstrates new prospects for the treatment of geriatric diabetes, but also provides a new direction for future therapeutic practice, especially in improving patients' quality of life and delaying disease progression.

## References

[1] Bellary S Kyrou I Brown JE Bailey CJ. Type 2 diabetes mellitus in older adults: clinical considerations and management. Nat Rev Endocrinol. (2021) 17:534–48. 10.1038/s41574-021-00512-2 34172940

[2] Cole JB Florez JC. Genetics of diabetes mellitus and diabetes complications. Nat Rev Nephrol. (2020) 16:377–90. 10.1038/s41581-020-0278-5 32398868 PMC9639302

[3] Zheng Y Ley SH Hu FB. Global aetiology and epidemiology of type 2 diabetes mellitus and its complications. Nat Rev Endocrinol. (2018) 14:88–98. 10.1038/nrendo.2017.151 29219149

[4] Armstrong DG Tan TW Boulton AJM Bus SA. Diabetic foot ulcers: a review. Jama. (2023) 330:62–75. 10.1001/jama.2023.10578 37395769 PMC10723802

[5] Srikanth V Sinclair AJ Hill-Briggs F Moran C Biessels GJ. Type 2 diabetes and cognitive dysfunction-towards effective management of both comorbidities. Lancet Diabetes Endocrinol. (2020) 8:535–45. 10.1016/S2213-8587(20)30118-2 32445740

